# A Detailed Family History of Myocardial Infarction and Risk of Myocardial Infarction – A Nationwide Cohort Study

**DOI:** 10.1371/journal.pone.0125896

**Published:** 2015-05-26

**Authors:** Mattis Flyvholm Ranthe, Jonathan Aavang Petersen, Henning Bundgaard, Jan Wohlfahrt, Mads Melbye, Heather A. Boyd

**Affiliations:** 1 Department of Epidemiology Research, Statens Serum Institut, Copenhagen, Denmark; 2 The Heart Center, Unit for Inherited Cardiac Diseases, Copenhagen University Hospital, Copenhagen, Denmark; University of Tampere, FINLAND

## Abstract

**Background:**

Family history of myocardial infarction (MI) is an independent risk factor for MI. Several genetic variants are associated with increased risk of MI and family history of MI in a first-degree relative doubles MI risk. However, although family history of MI is not a simple dichotomous risk factor, the impact of specific, detailed family histories has not received much attention, despite its high clinical relevance. We examined risk of MI by MIs in first- and second-degree relatives and by number and age of affected relatives.

**Methods and Findings:**

Using Danish national registers, we established a nationwide cohort of persons born between 1930 and 1992 with identifiable first- or second-degree relatives. Incident MIs in both cohort members and relatives aged ≥20 years were identified. We calculated incidence rate ratios (IRRs) for MI by family history of MI, by Poisson regression. In 4.4 million persons followed for 104 million person-years, we identified 128,384 incident MIs. IRRs with 95% confidence intervals [CIs] for MI by history of MI in 1, 2 or ≥3 first-degree relatives were 1.46 (1.42-1.49), 2.38 (2.22-2.56) and 3.58 (2.66-4.81), respectively. Corresponding estimates for second-degree relatives were 1.17 (1.05-1.30), 1.87 (1.46-2.38) and 2.18 (1.09-4.36). A history of MI in combinations of first- and second-degree relatives increased risks 1.8- to 7-fold in middle-aged persons (36 to 55 years). Estimates were robust to adjustment for diabetes, hypertension, dyslipidemia and use of cardiovascular medications.

**Conclusion:**

A detailed family history, particularly number of affected first- and second-degree relatives, contributes meaningfully to risk assessment, especially in middle-aged persons. Future studies should test for potential improvement of risk algorithm prediction using detailed family histories.

## Introduction

Cardiovascular disease in a first-degree relative confers an increase in cardiovascular disease risk, independent of established risk factors such as high blood pressure and elevated plasma lipid levels.[1–4] A recent combined analysis of 12 cohort studies found a combined relative risk of 1.6 for future events in persons with a first-degree relative with cardiovascular disease, compared with persons without an affected first-degree relative.[5] Genome-wide association studies continue to identify single nucleotide polymorphisms associated with coronary artery disease and/or myocardial infarction (MI).[6–8] The majority of these common genetic variants increase risk only modestly (< 20%) and the clinical usefulness of these genetic associations is limited and much debated; no genetic risk score has yet proven widely applicable in either primary or secondary prevention.[9] Family history is generally readily available to the clinician, and several studies have highlighted the importance of family history in risk prediction and possibly screening.[10–13] However, major risk scores such as the Framingham risk score and the European “SCORE” do not take family history into account, and thus far, few reclassification studies have evaluated the effect of integrating family history in cardiovascular outcome risk assessment.[14–17] Sivalapratnam et al. concluded that although family history was an independent risk factor for cardiovascular disease in a cohort of persons aged 40–79 years, adding family history as a yes/no variable to the Framingham algorithm did not improve risk classification.[16] In contrast, when Yeboah et al. evaluated risk prediction in intermediate-risk persons in a similar age range, of six independent risk markers, only family history (yes/no) and coronary calcium score were associated with hazards ratios ≥2, and the addition of family history to the Framingham algorithm resulted in better risk prediction, outdone only by coronary calcium score.[17] However, a major limitation of these studies was the inclusion of family history as a dichotomous variable, since previous cohort studies suggest that more detailed family histories are relevant to risk stratification.[1,18,19]

Number of affected relatives, degree of kinship and age at onset may help the clinician assess cardiovascular outcome risk more precisely, particularly in persons by current algorithms to be at intermediate risk of a future event and for whom prophylactic treatment is currently not indicated. The aim of this study was to examine the risk of MI in a nationwide cohort by family history of MI, with focus on details of number of affected relatives, degree of kinship, and age of relatives at time of MI.

## Methods

### Data sources

All data were obtained from Danish national health registers. Since 1968, the Danish Civil Registration System has assigned a unique personal identification number to every Danish resident, making it possible to link individual-level information across Danish registers.[20]

The Danish Family Relations Database, developed by researchers in the Department of Epidemiology Research at Statens Serum Institut, is based on parent-child links registered in the Civil Registration System. The Family Relations Database allows identification of family members for a given individual without requiring individual-level contact, permitting large-scale familial aggregation studies. All children and siblings are identifiable for >80% of individuals born in Denmark as early as 1930. For those born in 1950 or later, all first-degree relatives (parents, children and siblings) and half-siblings can be identified. Second-degree relatives (grandparents, grandchildren, aunts/uncles, nieces/nephews) can be identified for 90% of individuals born from 1985 onwards.

The National Patient Register contains information on inpatient diagnoses assigned since January 1, 1977 and outpatient diagnoses assigned from 1995 onwards.[21] Diagnoses are registered using International Classification of Disease (ICD) codes, with ICD-8 codes used until 1993, and ICD-10 codes used thereafter.

The National Diabetes Register is based on hospital diabetes diagnoses, filled prescriptions for diabetes treatment, primary care measurements of blood-glucose levels and reports of diabetic chiropody, from 1991 onwards. Validation of register information against a general practioner database found a sensitivity of 96% and a positive predective value of 89%.[22]

The Danish Register of Medicinal Product Statistics allows tracking of individual prescription drug histories from 1994 to the present.[23] Dispensed medications are identified by Anatomical Therapeutic Chemical classification (ATC) codes.

### Study Cohort, Ascertainment of Exposure Status and Follow-Up

Our study cohort included all residents of Denmark born since 1930 and alive on January 1 1977 or born thereafter, who were ≥20 years of age before the end of follow-up and had at least one identifiable relative. For each person, we identified all registered relatives using the Danish Family Relations Database and then used the National Patient Register to determine whether any relative had ever suffered a MI (ICD-8 codes 410.00–410.99, ICD-10 codes I21.00-I22.99). Family history of MI was handled as a time-dependent variable. A person was classified as having a family history of MI from the time an MI was first registered in a relative. If this occurred before the person’s 20^th^ birthday, that person was considered to have a family history of MI from the start of follow-up. If no relative was ever registered as having an MI, the person was considered not to have a family history of MI for the entire duration of follow-up. Family history was further categorized by degree of kinship, relative’s age at MI, and number of affected relatives. Cohort members were followed from January 1, 1977 or their 20^th^ birthday, whichever came later, until the first of the following events: 1) MI (ascertained the same way as in relatives) 2) death; 3) emigration; 4) designated “missing” in the Civil Registration System; or 5) May 31, 2012 (end of follow-up).

### Statistical Analysis

Using log-linear Poisson regression, we calculated incidence rate ratios (IRRs) comparing the rate of MI among persons with a relative who had had an MI (an “affected” relative) with the rate among persons with no affected relatives. In analyses of specific types of affected relatives, e.g. second-degree relatives, we compared only those individuals who had the specific type of relatives in question. This was done to reduce possible bias from incomplete identification of family members (due to the structure of the Civil Registration System and consequently the Danish Family Relations Database). Estimates were adjusted for attained age (5-year categories), sex of cohort member, and calendar period (5-year categories). We did not have sufficient information on the full cohort to classify individuals as high, intermediate or low risk according to traditional risk scoring; however age is one of the strongest risk factors for MI, and we also stratified our analyses by cohort member attained age (<36 years, 36–55 years, >55 years), with the middle group serving as a proxy for intermediate-risk individuals.

From 1994 to 2008, information on several of the risk factors used in traditional risk scoring was available. Therefore, in sub-analyses with follow-up limited to this period, we adjusted for hypertension (ICD-8 codes 400.09–402.99 and ICD-10 codes I10-I15 in the National Patient Register), use of cardiovascular medications (as a proxy for mild hypertension not registered in the National Patient Register, defined as filling two or more prescriptions for any of the following medications: diuretics [ATC code C03], beta-blockers [C07], calcium antagonists [C08], and medications with effects on the renin-angiotensin system [C09AA, C09B, C09CA, C09D]), diabetes (from the National Diabetes Register), and dyslipidemia (as a proxy for dyslipidemia, we identified persons filling ≥2 prescriptions for any lipid-modifying medication [ATC code C10]). These covariates were all handled as time-dependent variables.

To evaluate whether age at MI differed by family history, we tested whether there was an interaction between age and family history in the Poisson regression model; the significance of the interaction was evaluated using likelihood-ratio tests. All statistical analyses were conducted using PROC GENMOD in SAS software, version 9.3 (SAS Institute Inc., Cary, North Carolina).

## Ethical Considerations

The Department of Epidemiologic Research, Statens Serum Institut, has open approval from the Danish National Board of Health and the Danish Data Protection Agency to conduct epidemiological research studies using nationwide Danish registers such as those included in this study. Ethics Committee (local or national) approval to conduct this study was not needed since the study was register-based. We only used anonymized data (we removed personal information such as personal identification numbers and names), we only present data in aggregate and anonymous form, and we neither contacted any study sub-jects (cohort members and their families) nor required any active participation from them.

## Results

We followed a cohort of 4,445,255 persons for 104,135.666 person-years, for an average follow-up time of 23 years per person. During follow-up, we identified 128,384 first MIs. Table 1 shows the distribution of first MI by age, sex and proportion of persons with identifiable relatives. Among 121,022 persons with no history of MI in first-degree relatives (33,426 women and 87,596 men) but who experienced an MI during follow-up, the median age at MI was 57.0 years overall, 59.6 years for women and 56.5 years for men. In 7,362 persons (1,800 women and 5,562 men) with ≥1 first-degree relatives with MI who themselves experienced an MI during follow-up, the median age at MI was 51.0 years overall, 53.1 years for women and 49.8 years for men. These age differences by family history were statistically significant (P<0.001), both overall and by gender.

**Table 1:** Cohort Characteristics, Persons with Myocardial Infarction and follow-up by age, sex, and family history.

	Persons With MI (n = 128,384)		Duration of follow up, person years x 10^3^
**Age at Myocardial Infarction**	No. (%)		
20–29 years	800	(<1%)	30,381
30–39 years	5,706	(4%)	29,844
40–49 years	25,546	(20%)	26,505
50–59 years	42,503	(33%)	18,161
60–69 years	37,250	(29%)	9,461
70–79 years	16,060	(13%)	2,661
80-years	519	(<1%)	67
**Sex**			
Male	93,158	(73%)	59,139
Female	35,226	(27%)	57,942
**Number and proportion with identifiable relatives** ^**a**^			
First-degree relative^b^	114,141	(89%)	104,136
Second-degree relative^c^	75,765	(59%)	52,907
**Number and proportion with identifiable relatives with MI**			
First-degree relative with MI	7,362	(6%)	5,665
Second-degree relative with MI	420	(<1%)	2,333

Characteristics on 128,384 persons with myocardial infarction in a cohort of 4,445,255 persons born in 1930 or later and aged 20 years or more. The cohort was followed for 104,135,666 person-years from 1977 to 2012 in Denmark. Abbreviations: MI, Myocardial Infarction

^a^ Cohort members could contribute more than 1 type relative to the analyses; numbers add up to more than 128,384 (100%).

^b^ **First-degree**: parents, children and siblings.

^c^ **Second-degree**: grandparents, grandchildren, half-siblings, uncles, aunts, nieces and nephews.

Persons with a history of MI in ≥1 first-degree relatives were 1.52 times (95% confidence interval [CI] 1.48–1.55) as likely to suffer an MI as those without a history of MI in first-degree relatives. The magnitude of the IRRs increased dramatically with the number of affected first-degree relatives (Table 2). A similar pattern, albeit with smaller increases in risk, was seen for MIs in second-degree relatives (Table 2). When we considered only follow-up time for persons aged 36–55 years, the pattern remained the same: IRRs for a history of MI in one, two and three or more first-degree relatives were 1.50 (95% confidence interval [CI] 1.45–1.55), 2.79 (95% CI 2.56–3.04) and 4.20 (95% CI 2.86–6.16), respectively, while for a history of MI in second-degree relatives, the corresponding IRRs were 0.95 (95% CI 0.83–1.10), 1.65 (95% CI 1.21–2.24) and 1.69 (95% CI 0.64–4.52). To look at associations with even more comprehensive family histories, we combined information on MIs in both first- and second-degree relatives. For persons aged 36–55 years, the risks of MI increased as much as 7.5-fold (with ≥2 affected first-degree relatives and ≥2 affected second-degree relatives) (Table 3). (The corresponding estimates for the full cohort, and persons 20–25 years of age and >55 years of age are given as Supporting Information in S1 Table.)

**Table 2:** Incidence Rate Ratios of Myocardial Infarction by Number and Degree of Kinship.

Family history of MI in	Number of persons with MI and the specified family history	Duration of follow up, person years x 10^3^	Incidence rate ratios^a^ with 95% CIs
**First-degree relatives** ^**b**^			
None	106,779	94,470	1 (ref)
One	6,569	5,409	1.46 (1.42–1.49)
Two	749	249	2.38 (2.22–2.56)
Three or more	44	8	3.58 (2.66–4.81)
**Second-degree relatives** ^**c**^			
None	75,345	50,573	1 (ref)
One	347	1,995	1.17 (1.05–1.30)
Two	65	304	1.87 (1.46–2.38)
Three or more	8	34	2.18 (1.09–4.36)

Incidence rate ratios with 95% confidence intervals for myocardial infarction in all persons aged 20 years or more with one, two or three or more first- or second-degree relatives with myocardial infarction compared to persons with similar a number of relatives without myocardial infarction, follow-up from 1977 to 2012. Abbreviations: MI, Myocardial Infarction; CI, confidence interval

^a^ Incidence rate ratios are adjusted for age, sex and calendar period

^b & c^ For definitions of first- and second-degree relatives, see Table 1.

**Table 3:** Associations Between Complex Family Histories Of Myocardial Infarction And Myocardial Infarction Risk in Persons Aged 36-55y.

Number and degree of relatives with MI		Incidence rate ratios^a^with 95% CIs
**First-degree relatives** ^**b**^	**Second-degree relatives** ^**c**^	
None	None	1 (ref)
None	One or more	1.06 (0.91–1.23)
One	None	1.54 (1.49–1.59)
One	One	1.83 (1.40–2.40)
One	Two or more	2.46 (1.28–4.73)
Two or more	None	2.92 (2.68–3.18)
Two or more	One	3.71 (1.67–8.26)
Two or more	Two or more	7.52 (1.88–30.07)

Incidence rate ratios with 95% confidence intervals for myocardial infarction in persons aged 35 to 55 years with combinations of none, one or two or more first- and second-degree relatives with myocardial infarction, follow-up from 1977 to 2012. Abbreviations: MI, Myocardial Infarction; CI, confidence interval

^a^ Reference incidence rate are rates in those cohort members with a myocardial infarction and identifiable relatives of both first- and second-degree without myocardial infarction. Incidence rate ratios are adjusted for age, sex and calendar period.

^b & c^ For definitions of first- and second-degree relatives, see Table 1.

**Fig 1:**
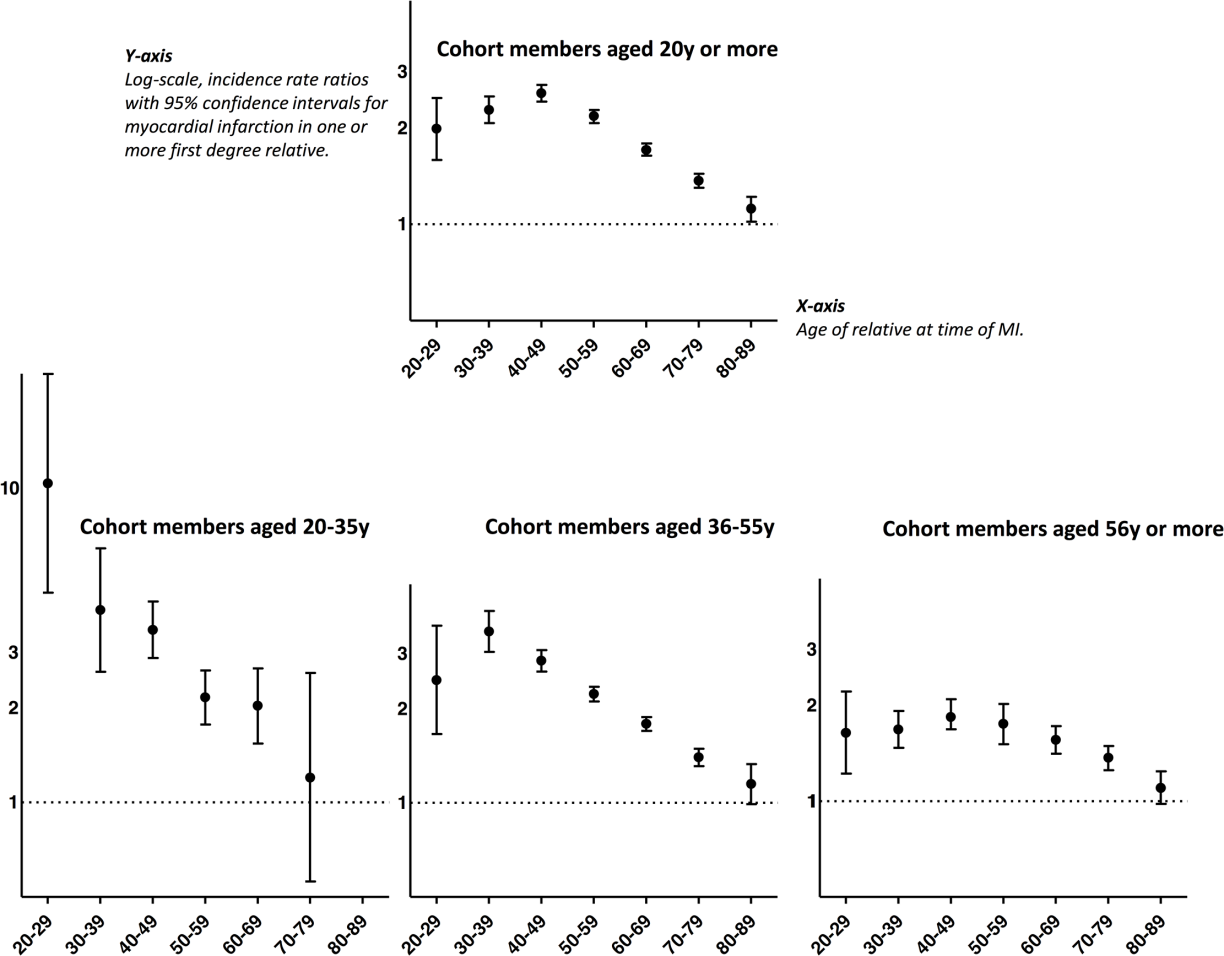
The four graphs in the figure illustrate incidence rate ratios (IRRs) with 95% confidence intervals (CIs) for myocardial infarction in persons with one or more first-degree relatives with myocardial infarction. The analyses were stratified by age of first-degree relative at time of myocardial infarction. The reference group for each IRR consists of persons with relatives in the given age group but without a history of MI among those relatives. Age of the relative at time of myocardial infarction is shown in decades on the x-axis, while the IRRs for myocardial infarction are shown on the y-axis. The upper graph shows the risk for all persons in the cohort ≥20 years of age at some point during the follow-up period. The three lower graphs shows the results from analyses restricted to follow-up time while cohort members were 20–35 years of age, 36–55 years of age and >55 years of age. In the analysis of persons aged 20–35 years, there were no first-degree relatives aged 80–89 years with a myocardial infarction.

IRR magnitudes depended both on the cohort member’s attained age and the age of the relative at MI (Fig 1). Younger age at MI in relatives was associated with higher MI risk. In the youngest cohort members (20–35 years of age), MI in the youngest relatives was associated with a 10-fold increase in MI risk. In cohort members aged 36–55 years risks, MI in relatives was associated with up to a 3-fold increase in MI risk (given relatives with MI 30–49 years of age), whereas in older persons (>55 years of age), increases in MI risk were 2-fold or less, regardless of a relative’s age at MI.

When we stratified our analyses by sex, our estimates remained unchanged (data not shown), with the association between a family history of MI and MI risk appearing to be the same in men and women.

In sub-analyses with follow-up only during the period 1994–2008 (when information on potential confounders was available), we followed 3,615,748 persons for 48,445,702 person-years and identified 70,536 MIs. The IRRs additionally adjusted for hypertension, diabetes, dyslipidemia and use of cardiovascular medication were practically unchanged compared to those only adjusted for age, sex and calendar period (Table 4).

**Table 4:** Minimal and Fully Adjusted Incidence Rate Ratios.

Family history of MI in	Incidence rate ratios with 95% CIs	
	Adjusted for age, sex and calendar period.	Adjusted for age, sex, calendar period, hypertension, diabetes, hyperlipidemia and use of cardiovascular medication.
**First-degree relatives** ^**a**^		
One	1.51 (1.46–1.56)	1.47 (1.42–1.52)
Two	2.60 (2.36–2.86)	2.42 (2.20–2.66)
Three or more	3.92 (2.58–5.95)	3.40 (2.24–5.17)
**Second-degree relatives** ^**b**^		
One	1.12 (0.96–1.30)	1.15 (0.99–1.33)
Two	1.85 (1.32–2.59)	1.89 (1.35–2.65)
Three or more	2.18 (0.82–5.80)	2.26 (0.85–6.03)

Incidence rate ratios with 95% confidence intervals for myocardial infarction in all persons aged 20 years or more with one, two or three or more first- or second-degree relatives with myocardial infarction compared to persons with similar a number of relatives without myocardial infarction. Follow-up from 1994 to 2010, minimally adjusted and more fully adjusted (with inclusion of other risk factors for myocardial infarction) IRRs are presented. Abbreviations: MI, Myocardial Infarction; CI, confidence interval

^a &^ ^b^ For definitions of first- and second-degree relatives, see Table 1.

## Discussion

Our study quantified how a detailed family history of MI affects relative risk of MI. Our findings provide new support for moving beyond dichotomous overall family history parameters and taking additional details into account (e.g. combining information on MI in first- and second-degree relatives, number of affected relatives, and age at MI) when assessing risk. Not unexpectedly, we showed unequivocally that the more MIs there have been in a family and the closer the degree of kinship, the more an individual’s risk of MI is increased. However, we also showed that MIs in second-degree relatives are associated with a considerable increase in MI risk, and that the age at which a relative had an MI has implications for the age at which a person is at greatest risk of themselves having an MI. Early MIs in relatives were associated with the greatest risks, especially in young persons. Interestingly, MIs in relatives >70 years of age were only weakly associated with risk, at any age, suggesting a limited value of history of MI in older relatives for risk prediction. More importantly, we found that in persons aged 36–55 years, a family history of MI was more strongly associated with MI risk than in persons >55 years of age.

Genome-wide association studies have identified more than 40 common genetic variants associated with coronary artery disease risk. [7,24] Each individual variant is only modestly associated with disease risk, with each variant estimated to increase risk 6–92%, but it is estimated that most individuals carry 20–40 risk alleles, suggesting that coronary artery disease and MI are usually polygenic outcomes. (In young persons (<35 years), familial dyslipidemia—which often exhibits a monogenic pattern of inheritance—is thought to contribute to most MIs.[25]) Our findings that MI risk increases with increasing number of affected relatives, increasing degree of kinship (i.e. of genetic relatedness) and decreasing age at MI in a relative are in agreement with a complex genetic background for coronary artery disease and MI.

Although recent genetic research has significantly improved our understanding of the hereditary aspects of cardiovascular disease and MI, it is highly improbable that we have identified more than a handful of the existing, relevant genetic variants, and risk prediction for these outcomes based on genetic testing is not yet possible.[24] Such testing would be especially useful in intermediate-risk individuals (persons with a 10-year cardiovascular disease risk exceeding 5% but less than 20%), since current guidelines do not recommend prophylactic pharmacological treatment for these persons. However, our findings suggest that a detailed family history of MI might serve as a reasonable proxy for genetic information and contribute to better risk stratification for MI, particularly in these ambiguous cases. Indeed, a family history of MI was more important in persons aged 35–55 years—who are typically regarded as at intermediate risk of a future event—than persons aged >55 years. A recent study of intermediate-risk individuals showed promising results when a dichotomous (yes/no) measure of family history of MI was added to the Framingham risk-scoring algorithm. [17] Our findings suggest that future studies should test whether the introduction of detailed family history, with more weight on younger (<50 years) and number of affected relatives, would improve the net reclassification of intermediate-risk individuals even more.

Prevention of MI includes primary prevention, i.e. identification of high-risk individuals and targeted reduction of risk by minimizing modifiable risk factors, and secondary prevention, i.e. minimizing risk factors in persons with existing cardiovascular disease. Primary prevention in unselected groups of the background population remains to be proven effective; in fact, both a recent Cochrane review and newly published Danish data suggest just the opposite.[26,27] A focus on prevention in high-risk groups might be more valuable and has been suggested.[14,26] In conjunction with the existing literature, our results indicate that persons with younger relatives with MI or many relatives with MI may comprise such a group of high-risk persons, even though traditional risk scoring considers them to be at low or intermediate risk.

A number of previous studies have examined the association between cardiovascular disease in up to two first-degree relatives, typically parents, and an individual’s own risk of adverse cardiovascular outcomes.[1–4,28] A recent analysis of 12 cohort studies yielded a relative risk of 1.60 (95% CI 1.44–1.77) for coronary heart disease given a positive family history in a first degree-relative,[5] which is similar to our overall estimate of 1.52 (95% CI 1.48–1.55). However, the relative risks estimated in some studies have been greater in magnitude than our estimates. Recall bias could inflate estimates in studies using self-reported family history.[1,2] Furthermore, differences in estimates from different studies are likely to reflect the varying age distributions of the study cohorts and different definitions of family history, both regarding the relatives in question and the types of cardiovascular events considered.[4,28] For example, a Danish study[28] found a 2- to 3-fold increase in risk of MI given MIs in parents or siblings, which are larger risks than we estimated for affected first-degree relatives. However, they only included persons born in or after 1954 (i.e. <58 years of age at the end of follow-up) and thus had a younger cohort than our overall cohort; their results compare favorably with our estimates for persons aged 20–55 years with relatives aged 20–59 years at time of MI.

In analyses on a sub-cohort with available information on risk factors included in traditional risk prediction algorithms (diabetes, hypertension, use of lipid-lowering medications, and use of other cardiovascular medications), adjustment for these factors did not change our conclusions. Thus, in line with studies examining the risk associated with parental MI, the increases in risk associated with a family history of MI was independent of the effects of other risk factors.[1–3,5] Information on body mass index and smoking is not available in the Danish national registers. However, previous studies have found that risks associated with family history were independent of smoking and body composition.[1–3]

The strengths of our study were the size of the cohort and number of person-years of follow-up, which allowed for very detailed family history analyses. Many previous studies have used self-reported family history, introducing the possibility of recall bias; we eliminated this possibility by using register-based MI diagnoses. MI diagnoses in the National Patient Register have been validated against Danish cases registered in the World Health Organization Monitoring Trends and Determinants in Cardiovascular Disease (MONICA) project and shown to have an excellent sensitivity (97%), a positive predictive value of 93.0% and a false positive rate of only 6.5%.[29] Furthermore, self-reported measures of family history in other studies have often included a combination of events of varying severity such as subtypes of coronary artery disease (MI, stable and unstable angina), stroke, and death. We used only hospital admissions for MI, a strict and validated endpoint subject to less misclassification and yielding more easily interpretable estimates. Finally, the Danish Family Relations Database allowed for identification of both first- and second-degree relatives for a cohort of unprecedented size, allowing for the detailed analysis of different types of family history.

## Conclusion

Cardiovascular disease risk, MI risk included, is influenced by both genetic and environmental factors. Information on family history is readily available and has implications for MI risk stratification: family history of MI is an important marker of increased MI risk, and particular weight should be placed on the number of affected first- and second-degree relatives and age of the relative at the time of MI. Our study suggests that such a detailed family history could be very useful in assessing MI risk, especially in persons aged 35–55 years.

## Supporting Information

S1 Table Associations between complex family histories of myocardial infarction and myocardial infarction risk, overall and by age group. (DOCX)
