# Supplementary material for: A Detailed Family History of Myocardial Infarction and Risk of Myocardial Infarction – A Nationwide Cohort Study
Source: PLoS One. 2015 May 26;10(5):e0125896. doi: 10.1371/journal.pone.0125896 (PMC4444238; doi:10.1371/journal.pone.0125896)
Supplement: S1 Table — (DOCX) [file pone.0125896.s001.docx]

| **Supporting Table 1. A Detailed Family History of Myocardial Infarction**  **Associations between complex family histories of myocardial infarction and myocardial infarction risk, overall and by age group.**  **Incidence rate ratios with 95% confidence intervals for myocardial infarction in persons aged 20 year or more, and 20 to 35 years 36 to 55 years, and 56 years or more with combinations of none, one or two or more first- and second-degree relatives with myocardial infarction, follow-up from 1977 to 2012.** | | | | | |
| --- | --- | --- | --- | --- | --- |
| Age of cohort members | Number and degree of relatives with MI | | Number of persons with MI and the specified family history | Duration of follow up,  person years x 10^3^ | Incidence rate ratios^a^  with 95% CIs |
|  | **First-degree relatives^b^** | **Second-degree relatives^c^** |  |  |  |
| Whole cohort  (≥20 years of age) | None | None | 106,459 | 96,277 | 1 (ref) |
|  | None | One or more | 320 | 2,193 | 1.32 (1.18-1.48) |
|  | One | None | 6,480 | 5,272 | 1.49 (1.45-1.53) |
|  | One | One | 76 | 113 | 1.98 (1.58-2.48) |
|  | One | Two or more | 13 | 22 | 2.92 (1.69-5.03) |
|  | Two or more | None | 782 | 252 | 2.49 (2.32-2.68) |
|  | Two or more | One | 8 | 3 | 2.86 (1.43-5.71) |
|  | Two or more | Two or more | 3 | <1^e^ | 7.42 (2.39-23.0) |
|  |  |  |  |  |  |
| 20-35 years | None | None | 1,805 | 35,963 | 1 (ref) |
|  | None | One or more | 102 | 1,844 | 1.20 (0.98-1.48) |
|  | One | None | 241 | 1,578 | 2.33 (2.03-2.66) |
|  | One | One | 13 | 66 | 3.27 (1.89-5.65) |
|  | One | Two or more | 3 | 16 | 3.52 (1.13-10.9) |
|  | Two or more | Any ^d^ | 20 | 29 | 8.82 (5.67,13.72) |
|  |  |  |  |  |  |
| 36-55 years | None | None | 39,897 | 40,566 | 1 (ref) |
|  | None | One or more | 176 | 298 | 1.06 ( 0.91-1.23) |
|  | One | None | 4,211 | 3,039 | 1.54 ( 1.49-1.59) |
|  | One | One | 53 | 41 | 1.83 ( 1.40-2.40) |
|  | One | Two or more | 9 | 6 | 2.46 ( 1.28-4.73) |
|  | Two or more | None | 542 | 177 | 2.92 ( 2.68-3.18) |
|  | Two or more | One | 6 | 2 | 3.71 ( 1.67-8.26) |
|  | Two or more | Two or more | 2 | <1^e^ | 7.52 ( 1.88-30.07) |
| >55 years | None | None | 60,758 | 15,729 | 1 (ref) |
|  | None | One or more | 25 | 6 | 1.40 (0.95-2.07) |
|  | One | None | 1,750 | 411 | 1.25 (1.19-1.31) |
|  | One | One | 10 | 2 | 1.54 (0.83-2.85) |
|  | One | Two or more | - | <1^e^ | - |
|  | Two or more | None | 191 | 191 | 1.65 (1.43-1.90) |
|  | Two or more | One | 2 | <1^e^ | 1.79 (0.45-7.18) |
|  | Two or more | Two or more | 1 | <1^e^ | 7.68 (1.08-54.5) |
|  |  |  |  |  |  |
| Abbreviations: MI, Myocardial Infarction; CI, confidence interval  ^a^ Reference incidence rate are rates in those cohort members with an MI and identifiable relatives of both first- and second-degree without MI. Incidence rate ratios are adjusted for age, sex and calendar period  ^b^ **First-degree:** parents, children and siblings.  ^c^ **Second-degree:** grandparents, grandchildren, half-siblings, uncles, aunts, nieces and nephews  ^d^ There were no persons aged 20-35 years with MI and two or more first-degree relatives with MI and MIs in second-degree relatives. For this reason, all histories of affected second-degree relatives in those with two or more affected first-degree relative were collapsed into one group.  ^e^ There were less than 1,000 person-years of follow-up in this group. | | | | | |
